# Glassy carbon microneedles—new transdermal drug delivery device derived from a scalable C-MEMS process

**DOI:** 10.1038/s41378-018-0039-9

**Published:** 2018-12-17

**Authors:** Richa Mishra, Bidhan Pramanick, Tapas Kumar Maiti, Tarun Kanti Bhattacharyya

**Affiliations:** 1 Advanced Technology Development Center, IIT Kharagpur, Kharagpur, West Bengal 721302 India; 2 Department of Mechanical Engineering, IIT Kharagpur, Kharagpur, West Bengal 721302 India; 3 Biotechnology Department, IIT Kharagpur, Kharagpur, West Bengal 721302 India; 4 Department of Electronics and Electrical Communication Engineering, IIT Kharagpur, Kharagpur, West Bengal 721302 India; 5 Present Address: School of Electrical Sciences, IIT Goa, Ponda, Goa 403401 India

## Abstract

Because carbon is the basic element of all life forms and has been successfully applied as a material for medical applications, it is desirable to investigate carbon for drug delivery applications, as well. In this work, we report the fabrication of a hollow carbon microneedle array with flow channels using a conventional carbon-microelectromechanical system (C-MEMS) process. This process utilizes the scalable and irreversible step of pyrolysis, where prepatterned SU-8 microneedles (precursor) are converted to glassy carbon structures in an inert atmosphere at high temperature (900 °C) while retaining their original shape upon shrinkage. Once converted to glassy carbon, the microneedles inherit the unique properties of hardness, biocompatibility, and thermal and chemical resistance associated with this material. A comparative study of hardness and Young’s modulus for carbon microneedles and SU-8 microneedles was performed to evaluate the increased strength of the microneedles induced by the C-MEMS process steps. Structural shrinkage of the carbon microneedles upon pyrolysis was observed and estimated. Material characterizations including energy-dispersive X-ray spectroscopy (EDX) and Raman spectroscopy were carried out to estimate the atomic percentage of carbon in the microneedle structure and its crystalline nature, respectively. Our investigations confirm that the microneedles are glassy in nature. Compression and bending tests were also performed to determine the maximum forces that the carbon microneedles can withstand, and it was found that these forces were approximately two orders of magnitude higher than the resistive forces presented by skin. A microneedle array was inserted into mouse skin multiple times and was successfully removed without the breakage of any microneedles.

## Microneedles: Ultra-hard carbon gets under the skin

A technique for turning polymer films into a fracture-resistant, glassy form of carbon could hold promise for drug delivery. Microneedles are devices designed to inject or infuse medications into the body relatively painlessly by penetrating just below the skin. Richa Mishra and colleagues from IIT Kharagpur in India have now developed a biocompatible microneedle that remains intact after multiple insertions into mouse skin. The team’s approach uses photolithography to generate an array of vertically oriented hollow tubes, made from the epoxy-based polymer SU-8, on a silicon substrate. Heat treatments in an oxygen-free oven sharpened the microstructures into needle-like shapes composed almost exclusively of randomly ordered carbon atoms. Nanoindentation experiments revealed the glassy carbon structure could exert forces strong enough to pierce human skin with minimal risk of breakage.

## Introduction

Carbon is an intriguing material used for various applications, including medical technology. As a major advantage of carbon over silicon or other materials, carbon is available in various allotropes with unique physicochemical properties, which allows researchers to tailor its properties for unique applications^1^. The use of carbon in the medical field is not new; carbon has long been used for orthopedic joints, and carbon fibers and composites are used for orthopedic surgeries, surgical instruments, and other applications^2–5^. Furthermore, carbon has been studied for applications such as artificial heart valves and ear drum repair material^6^. Carbon nanofiber-reinforced composites are promising biomaterials and have been applied in cancer treatment^7^. Moreover, three-dimensional scaffold-like structure generation from carbon nanofibers can induce bone tissue regeneration^8^. Carbon nanotubes (CNTs) are another promising biomaterial with safe clinical use^9^. In this work, the use of carbon structures as hollow microneedles (MNs) in drug delivery is reported.

**Fig. 1:**
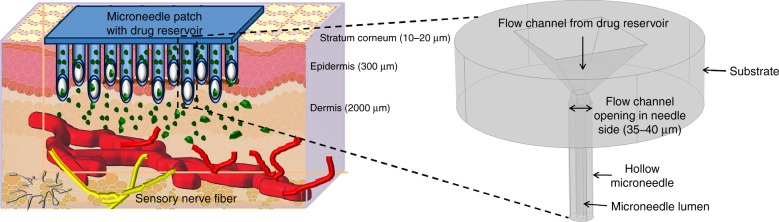
An illustration of drug delivery by hollow MNs. Hollow MNs puncture the skin to reach above the pain-sensing nerves in the transdermal region of the skin and painlessly release the drug through suitable actuation methods. The figure in the outset shows a magnified view of the microneedle structure proposed in this work

However, another interesting area in which the tailored properties of carbon can have a considerable impact in the biomedical device industry is the field of transdermal drug delivery using MNs. MNs are micron-sized needles that puncture the skin and deliver drugs to transdermal region of the skin, either by manual pressing or by a micropump^10^. To deliver the drug through the skin in the epidermis region, the MNs should be sufficiently long to effectively deliver the drugs while being short enough to avoid causing pain^11,12^. Microneedles have been researched for more than four decades; currently, researchers are exploring methods for selecting materials and methods that can be translated to commercial products in the minimum time^13–15^. With the established efficacy and painlessness of MNs, a plethora of research has been directed toward fabricating MNs with different shapes, sizes, and materials. The search for potential materials, dimensions, and designs for MNs has been centered on the requirements of biocompatibility, high strength to break through the stratum corneum (the outermost layer of the skin) and controlled drug delivery. Initially, research focused on silicon, as it was widely accepted by the microelectronics and MEMS industry;^16,17^ later, other materials such as steel, titanium, and nickel were used through either subtractive or additive processes^18^. Toward the last decade, MNs fabricated from polymers received more attention than other materials due to their flexibility in manufacturing and customizable mechanical properties^19,20^. MN materials may be dissolvable, swellable or biodegradable, and the structures may be in-plane or out-of-plane, solid or hollow, coated, dissolving, or hydrogel-forming^11,16^. The disadvantages of solid, coated, dissolving, or hydrogel-forming MNs include their ability to deliver only a limited amount of drugs. Hollow MNs can painlessly deliver larger quantities of drugs with a controllable flow rate (Fig. 1). Additionally, hollow MNs can be attached to a drug reservoir, and micropumps can be employed to push the drug through the MNs^10^. One such proposed design is shown in Fig. 1 ^21^.

In drug administration through MNs, the MN tip may break after insertion into the skin or during MN retraction. These broken tips should not cause negative immunogenic effects^22,23^. Hence, biocompatibility of the MN material remains a prime concern^24^. The materials used for MN fabrication include metals, silicon, glass, ceramics, and polymers such as PLGA and SU-8^18^. In spite of its wide use in earlier days, the biocompatibility of silicon is debatable^22,25^. Among polymers, the negative photoresist SU-8 is often selected as a potential material for MN fabrication based on its high cross-linking strength, biocompatibility, low-cost, light-induced polymerization, and microelectronic industry process compatibility^26^. SU-8 consists of bisphenol (a novolac epoxy resin) dissolved in gamma-butyrolactone, GBL or cyclopentanone, and a photoinitiator that composes 10% wt of the resin mass^27^. Recent works in carbon-microelectromechanical system (C-MEMS)-based glassy carbon microneedles (CMNs) have provided a milestone for biocompatible, high-strength, and patternable CMNs^28,29^. Conversion of the SU-8 polymer precursor to glassy CMNs by pyrolysis paves the way for high-strength MNs comprised of carbon, which is the building block of organic life, via a low-cost, reliable, and simple process^30–32^. Schueller et al. first demonstrated a practical carbon microfabrication technique using organic polymer precursors^33^. Since then, C-MEMS-based structures have been used for a variety of applications including batteries^34^, fuel cells^35^, dielectrophoresis^36^, supercapacitors^37^, scaffolds^38^, carbon nanowires^39^, transmission electron microscopy grids^40^, electroosmotic micropumps^41^, and gas sensors^42^.

The C-MEMS process includes spin coating of a carbon precursor photoresist, a soft bake, UV exposure, a postbake, and development^1,43^. After photolithography, carbon structures are obtained through a pyrolysis process.

In this work, we introduce the fabrication of an out-of-plane hollow carbon microneedle array obtained from an SU-8 microneedle (SMN) precursor. The MNs are pyrolyzed in an inert atmosphere at high temperature (900 °C), and the SU-8 is pyrolyzed to yield glassy carbon structures. Structural shrinkage is observed and estimated. The CMNs were characterized with respect to their mechanical properties such as hardness and Young’s modulus for comparison with their precursor counterparts; the results indicate that the CMNs are superior. Compression and bending tests were performed to determine the maximum compression and bending force that the CMNs can withstand, and it was found that these forces were approximately two orders of magnitude higher than the resistive forces presented by skin. The CMNs were inserted into mouse skin multiple times and successfully removed, without the breakage of any CMNs.

## Results and discussion

**Fig. 2:**
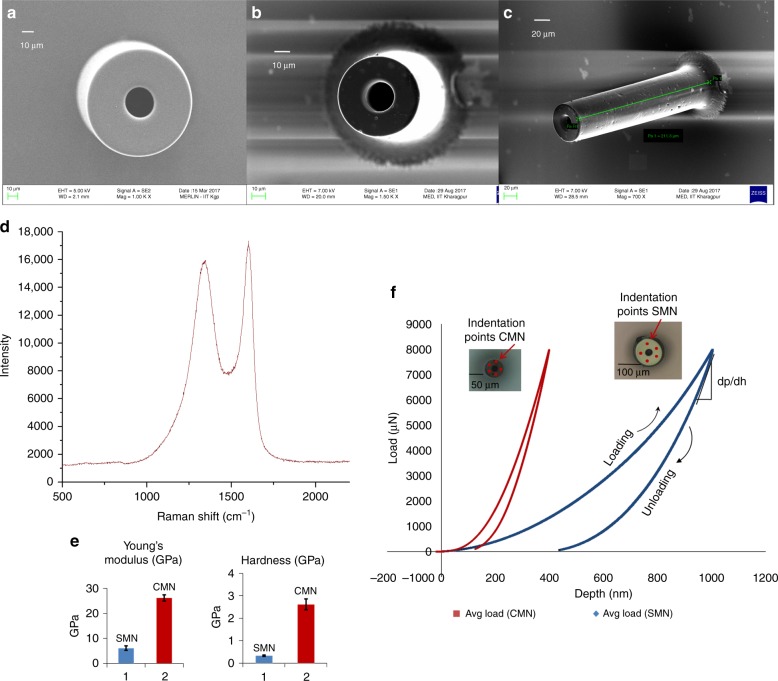
**a** Scanning electron micrograph of an SMN (outer diameter 100 μm, inner diameter 50 μm). **b** Corresponding pyrolyzed MN. **c** Tilted view of the same CMN. **d** Raman spectrum of the carbon microneedle. **e** Comparison of Young’s modulus and hardness for the SU-8 and carbon MNs. **f** Load vs. displacement data for an SMN and corresponding pyrolyzed CMN

SEM images were obtained for a single SMN (precursor) and the corresponding pyrolyzed CMN structure, and their dimensions were noted. The dimensions of the SMN and the corresponding dimensional changes observed in the CMN for different microneedle geometries are reported in Table 1. For the microneedle shown in Fig. 2, the outer diameter of 100 μm decreased to 59.77 μm, while the inner diameter of 50 μm decreased to 25.41 μm after pyrolysis. The microneedle wall thickness decreased from 25 μm to 16.77 μm.

**Table 1:** Shrinkage of microneedle dimensions upon pyrolysis

Sample Name	OD SMN	OD CMN	LD ratio	ID SMN	ID CMN	LD ratio	SW thickness	CW thickness	LD ratio
A	100	59.77	0.4	40	14.84	0.62	30	22.46	0.25
B	100	58.98	0.41	50	25.44	0.49	25	16.77	0.32
C	100	56.64	0.43	60	25.78	0.57	20	15.43	0.22
D	100	53.91	0.46	70	34.8	0.5	15	9.55	0.36
E	100	52.17	0.47	80	38.28	0.5	10	6.94	0.306
F	100	53	0.47	90	38.67	0.57	5	4.66	0.06

*OD* outer diameter, *LD* lateral decrease, *ID* inner diameter, *SW* SMN wall, *CW* CMN wall

On average, a shrinkage of 43% occurs for the outer diameter while the inner diameter decreases by 54%. The microneedle wall thickness is reduced via pyrolysis by an average of 29%. The EDX results presented in Table S1 (Supporting Document) show the atomic percentage of elements present in the SU-8 microneedles (SMNs) and CMNs. Notably, the atomic percentage of carbon increases from 76.35 to 94.46 when the SU-8 microneedle is transformed to a carbon microneedle. The absence of nitrogen in the carbon microneedle indicates that all of the nitrogen (atomic percentage 6.29) is removed by the heating process. It can be assumed from the change in the atomic percentage of oxygen (16.54%–4.93%) that most of the oxygen is removed from the MN structure, with only a small amount remaining. The identified oxygen present in the CMN arises from the contributions of the CMN structure and the Si/SiO_2_ substrate surface^44^.

The optimum dimensions for an SMN precursor that yields a CMN of dimensions appropriate for our drug delivery design (Fig. 1) upon shrinkage correspond to sample D. Hence, the sample D SMN (outer diameter of 100 μm, inner diameter of 70 μm) and the corresponding CMN (outer diameter of ~55 μm, inner diameter of 35 μm) were used for all comparative characterizations discussed henceforth.

Raman spectroscopy results of the MNs are presented in Fig. 2d. Two peaks corresponding to the D- and G-band are centered at ~1350 cm^−1^ and 1594 cm^−1^, respectively. The crystallinity of the structure is defined by the intensity ratio (I_D_/I_G_) of these bands^38^. The purpose of this characterization was to verify the nature of the obtained carbon. The results agree well with previous works^30^, and it was found that the microstructure of the carbon obtained here is glassy in nature.

Typical load displacement data were obtained for a Berkovich indentor as it was driven into and pulled out of the SU-8 and carbon microstructures. The results are shown in Fig. 2e, f. The hardness and modulus of elasticity were calculated for five points using the Oliver Pharr model and are shown in Fig. 2e. The hysteresis between the loading and unloading curve indicates the energy dissipated by indentation. The indentation SMN (outer diameter of 100 μm and inner diameter of 40 μm) indicate a hard elastoplastic behavior with a hysteresis loop between the loading and unloading curves. A quantitative analysis of the nanoindentation tests reveals a hardness of 0.33 GPa and a Young’s modulus of 5.52 GPa. Upon conversion of the SMN to a CMN via pyrolysis, the indentation curve changes to that of a highly elastic material (Fig. 2f). The hardness increases to 2.62 GPa (eightfold), and Young’s modulus increases to 26.97 GPa (4.8-fold).

For hollow MNs to be used as a biological interface for transdermal drug delivery, their maximum bending and compression forces should be higher than the skin insertion force so that they do not break during skin insertion.

As an MN is inserted, it experiences resistive forces from human skin, among which compression and bending forces are dominant. To successfully penetrate human skin, the applied force should be greater than this opposing force. The force required for puncturing skin is given by

1 $$F_{\rm {Skin}} = P_{\rm {Puncturing}}{\,} A$$

Typically, P_Puncturing_ is 3.18 MPa, but after the skin is punctured, the resistive force decreases drastically, dropping to 1.6 MPa^45–48^. If we consider a simple hollow cylindrical CMN with an outer diameter of 55 μm and an inner diameter of 20 μm, the cross-sectional area is given by

2 $$A = {\rm {\uppi}} \left( {R_o^2 - R_i^2} \right).$$

where R_o_ and R_i_ are the outer and inner radius, respectively. The bending forces acting on the microneedle during skin insertion are smaller (~1/100 of the compression forces presented by skin). The resistive force presented by skin to the CMN (sample D) is given in Table S2 (Supporting Document).

**Fig. 3:**
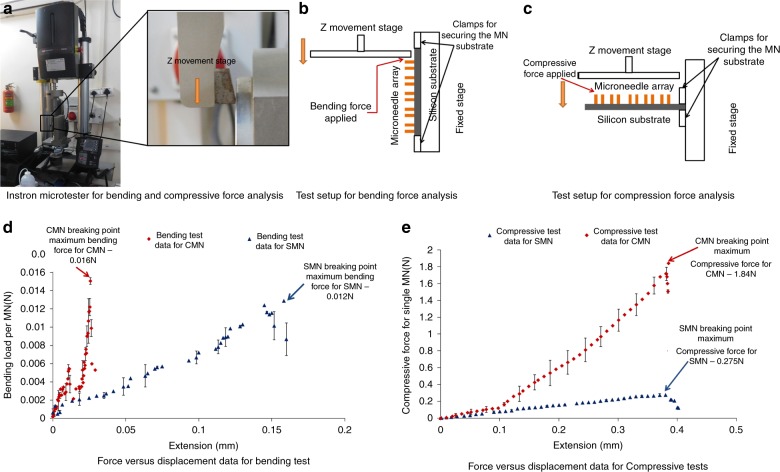
**a** Photograph of the Instron microtester employed for bending and compressive force analysis. **b** Test setup for bending force analysis. **c** Test setup for compression force analysis. **d** Force vs. displacement results from the bending test. **e** Force vs. displacement results from the compression test

The MN array was loaded on an Instron microtester (Instron, USA) (Fig. 3a). A metal plate was driven toward the MNs (both SMN and CMN) until they broke. For bending tests, two sides of a wafer were held securely by clamps. For compression tests, the sample was loaded on a 90° rotated T-shaped structure composed of aluminum that was held securely between the clamps; the MN array substrate was attached to this fixture using glue. The compressive and bending tests were performed on four samples each of SMNs (100-μm outer diameter and 70-μm inner diameter) and CMNs (55-μm outer diameter and 35-μm inner diameter). The compression and bending test setup and results are shown in Fig. 3b, c and Fig. 3d, e, respectively. The needles will not break if the applied load is below the maximum compressive or bending force. As the z stage applies an increased load, the needles eventually break. The sharp drop in the curves (Fig. 3d, e) marks the fracture point^49^. In the compression tests, when the MNs were parallel to the metal plate, the plate pressed and broke 100 (10 × 10) MNs together. In the bending tests, the MNs were perpendicular to the metal plate, which bent and broke 10 MNs at once. Hence, the recorded load was divided by 100 for the compression tests and by 10 for the bending tests. These tests were repeated to obtain an average data plot. In Fig. 3c, there is a slope variation at 0.1 mm, which might be attributed to slight initial bending of the aluminum fixture. The actual compression of the MNs started from an extension of 0.1 mm. In the bending tests, the CMNs showed a higher bending strength than the SMNs (~1.33-fold higher). A similar trend was observed for the compression tests, where the CMNs showed a higher compressive strength (~6.7-fold higher) than their precursor. Thus, these CMNs are much stronger, enabling them to overcome the resistive forces presented by skin to successfully pierce the skin (Table S2).

**Fig. 4:**
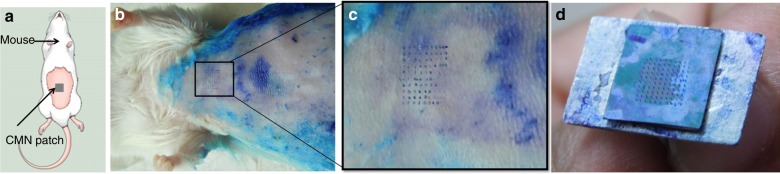
**a** Schematic of the MN insertion test on mice. **b** Biological insertion test performed on 6- to 8-week-old Swiss Albino mice. **c** Magnified view of the skin area pierced by the CMNs. **d** Intact array of 10 × 10 after multiple insertions

A mechanical interaction characterization of the hollow MNs with mouse skin was performed by driving the microneedle array perpendicularly against freshly excised mouse skin (Fig. 4a). The microneedle array was dipped in methylene blue and brought into contact with mouse skin; the array was pressed against the skin and then slowly withdrawn. This procedure was repeated multiple times. Upon visual examination, the microneedle marks of methylene blue were faintly visible on the mouse. Some of the MN marks could not be seen due to the uneven surface available for MN insertion. The microneedle spacing is sufficiently large to prevent the “bed of nails” effect, with a 500-μm array spacing^22^. The microneedle array was examined to verify that all of the MNs remained intact after insertion at different locations on the mouse skin, even after 15 insertions (Fig. 4). The fabricated CMNs with a 500-μm array spacing successfully penetrated the mouse skin surface and remained intact after retraction.

**Fig. 5:**
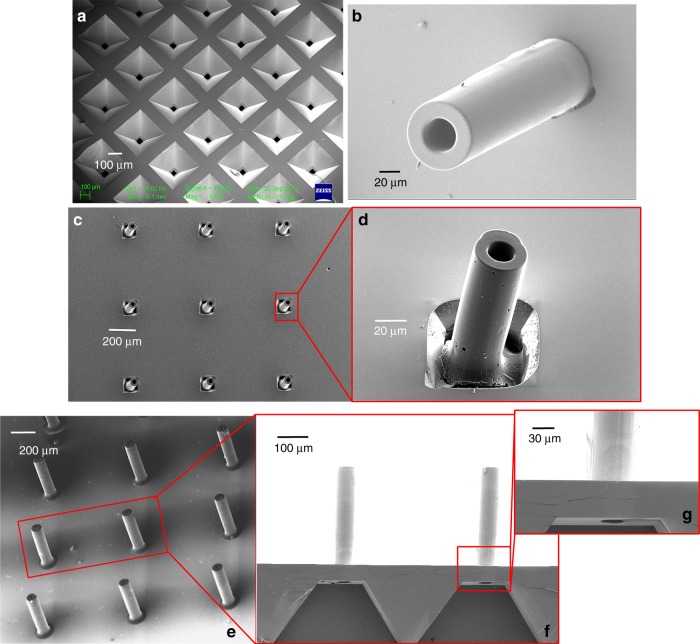
**a** Etched microfluidic conduit in silicon through which the drug flows from the drug reservoir to the CMN. **b** SMN fabricated on a microfluidic conduit backside of the image shown in **a**. **c** CMN array formed after pyrolysis. **d** Magnified view of a CMN. **e** Optimized CMNs aligned on etched microfluidic ports on a silicon wafer

The results for these MNs substantiate the use of glassy carbon MNs for transdermal drug delivery. To fabricate MNs as a device that can be readily connected to a drug reservoir with a suitable actuation method, hollow MNs should be fabricated on a substrate with flow channels. Figure 5a shows microfluidic conduits etched in silicon by wet chemical etching. SMNs were fabricated by direct laser writing, which precisely aligned them on the etched conduits (40 μm on the MN side) in silicon, as shown in Fig. 5b. The pyrolysis process was applied to the SMNs, and the resulting CMNs are shown in Fig. 5c. Figure 5d shows a magnified view of a CMN fabricated on the etched silicon substrate. In the process of conversion of SMNs to CMNs, the MNs nearly detach from the substrate. Hence, the dimensions of the flow channel were reduced based on the shrinkage ratio, and the CMNs cover the flow channel to prevent any fluid leakage or MN breakage. Therefore, by optimizing the flow channel dimensions, the SMNs (with the parameters of sample D) yielded CMNs that completely covered the fluidic channels (Fig. 5e). Magnified images of the CMNs and the underlying flow channel are shown in Fig. 5f, g.

**Fig. 6:**
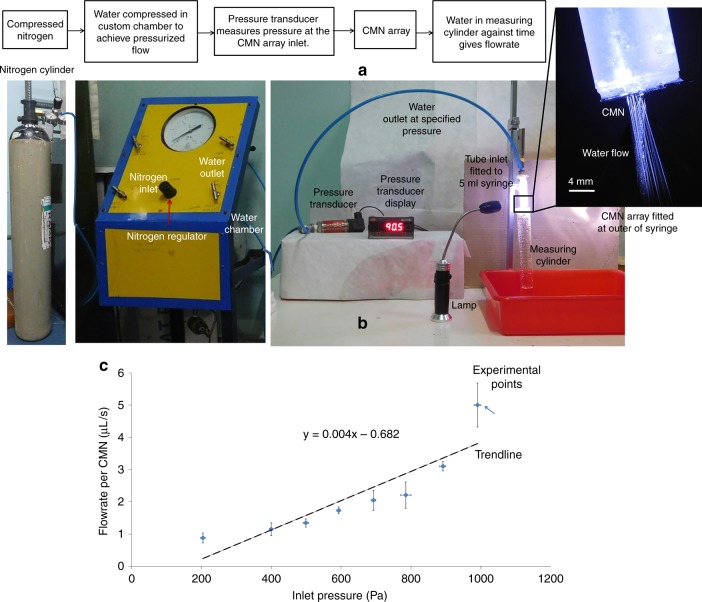
**a** Test setup for flow rate measurement. **b** Flow rate per microneedle at different inlet pressures

For flow rate measurements, a CMN array on a silicon substrate is attached to a 5-ml syringe, as shown in Fig. 6a. A customized chamber containing DI water is used, where pressurized nitrogen causes the DI water to flow through a tube connected to the syringe inlet. An Abus PT series pressure transducer monitors the pressure difference near the syringe inlet. In this setup, DI water can pass through the CMN array at a given pressure difference. The water is collected in a calibrated beaker, and the volume dispensed over time is recorded. This information gives the flow rate of the DI water through the CMNs for a given pressure difference. The test setup for flow rate measurements of MNs is shown in Fig. 6a, and a graph of the flow rate versus pressure for individual MNs is plotted in Fig. 6b. Measurements were acquired for three points corresponding to each pressure difference point, and the average flow rate is plotted. We note that an increased pressure corresponds to an increased flow rate while inducing a greater driving force. Overall, the results show that drug delivery can be controlled by controlling the inlet pressure. Hence, a known quantity of liquid drug formulation can be delivered to the human body. For a low pressure difference (< 100 Pa), initial droplets exited from the CMNs, but at higher pressures, a jet of water exited from the CMNs. The jet exiting from the MNs could be easily seen, and jets of water were observed exiting almost all of the MNs. Thus, 100% of MNs were fabricated with clear lumen to allow water to flow through them.

## Materials and methods

### Design

It is important to consider the appropriate dimensions of the MN for painless application. The microneedle length required to overcome the resistive forces presented by skin and to deliver the drug in the dendritic-cell-rich dermis well above the nerve cells is in the range of 200 μm–900 μm. The width of the needle must offer a high fracture force with little tissue damage (160 μm–800 μm) and clog-free lumen (typically more than 35 μm)^50^. Moreover, upon pyrolysis, structural shrinkage leads to a change in the microneedle dimensions; therefore, MNs of different geometries were considered for a parametric study on their dimensions. We considered six different designs for this work, keeping the outer diameter constant at 100 μm for all of the designs and varying the inner diameter from 40 μm to 90 μm in 10-μm increments. A list of the dimension parameters is presented in Table S3 (Supporting Document).

### Hollow carbon microneedles

**Fig. 7:**
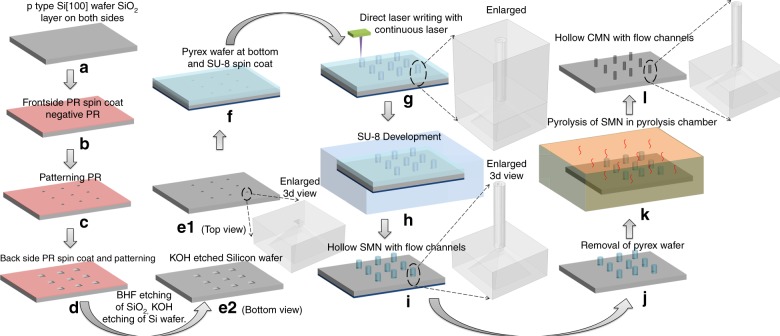
Process steps for the conversion of SMNs to CMNs by pyrolysis. The SMN structures shrink while retaining their overall geometry

The C-MEMS process was adapted for the fabrication of CMNsby patterning SU-8 microneedles (SMNs) via the direct laser writing technique^41^ and converting them to glassy CMN via pyrolysis. A schematic of the fabrication process steps for obtaining hollow CMNs is shown in Fig. 7.

### Fabrication of microfluidic ports

Microfluidic ports were etched in a silicon wafer, similar to our earlier work^51^. For this step, a 2” silicon (p-type, 100 plane) wafer was used as the substrate; the wafer was cleaned by the piranha method (H_2_SO_4_ and H_2_O_2_ at a ratio of 1:1) for 30 min and blown dry with nitrogen. Then, after a dehydration bake in an oven for 30 min, an SiO_2_ layer with a thickness of ~ 1 μm was formed on the silicon using the dry-wet-dry oxidation technique in a oxidation furnace (Tempress, Netherlands). The wafer was spin-coated with the negative photoresist (PR) HNR120 (Fujifilm, Japan) at 500 rpm for 5 s and at 3000 rpm for 20 s. After a prebake step for 30 min in an oven at 90 °C, the wafer was exposed in a Karl Suss MA6 (Italy) mask aligner (UV 365-nm wavelength) for 6.5 s using a square pattern for potassium hydroxide (KOH) etching of silicon. After the exposure, the sample was developed in WNRD developer (Fujifilm, Japan) and rinsed with n-butyl-acetate. The sample was postbaked at 120 °C for 30 min with the resist pattern on one side of the wafer. A similar procedure was carried out to pattern the second side of the wafer, with 400-μm squares aligned to the other side. The silicon dioxide layer was removed from the unprotected region by etching with buffered hydrofluoric acid (BHF) for 4–5 min. A piranha solution (H_2_SO_4_ and H_2_O_2_ at a ratio of 1:1) was applied for 30 min to remove the crosslinked photoresist layer. Then, a KOH (40% by weight) solution was prepared at 70 °C, and flow channels were etched in silicon at an etch rate of 0.9 μm per min. The sample was rinsed and dried and was then slowly placed on a 2” Pyrex wafer to prevent SU-8 from spilling from the etched holes in subsequent steps and to provide mechanical strength to the sample during handling.

### Fabrication by direct laser writing

SU-8 2150 (MicroChem, USA) was spun first at 500 rpm for 10 s to evenly spread the viscous photoresist and then at 1000 rpm for 30 s to obtain the desired thickness of 500 μm. The samples were soft-baked on a hotplate at 65 °C for 10 min and then at 95 °C for 120 min. A highly coherent laser beam from a laser writer system (helium–cadmium laser at 325 nm, Microtech LW-405B, Italy, laser spot size of 5 μm) was used for photopolymerization of the SU-8 at an intensity of 1277 mJ/cm^2^. The tightly focused helium–cadmium laser beam (365 nm) used for DLW has a lower diffraction-related loss than the UV photolithography technique and prevents the problem of undesired SU-8 cross-linking^27,52^. The hollow MNs on the flow channels of the substrate using the laser writer alignment technique. In this technique, two pattern points (A and B), whose design coordinates are known, are found on the wafer. Once these points are marked, the design file orients and rotates itself to match the design points A and B to the actual pattern points A and B on the wafer, and the laser exposure occurs in the SU-8 layer exactly above the patterns on the etched substrate. A postexposure bake was carried out for 5 min at 65 °C and for 30 min at 95 °C on a hotplate. After the postbake step, the Pyrex wafer was removed while the SU-8 was still hot and molten, and the sample was gradually cooled. The sample was developed in the SU-8 developer solution (MicroChem, USA). After development, the sample was rinsed in isopropyl alcohol and then slowly lightly blown with dry nitrogen.

### Pyrolysis

The fabricated samples were pyrolyzed in a furnace (Tempress, Netherlands) pressurized under an inert gas flow of N_2_^53^. The following temperature profile was used: a temperature increase from room temperature to 300 °C at a rate of 5 °C/min, a constant temperature for 60 min, a temperature increase from 300 °C to 900 °C at a rate of 5 °C/min, a constant temperature of 900 °C for 60 min, and a temperature decrease to room temperature at a rate of 10 °C/min.

### Characterization

SEM (EVO 18, ZEISS, Germany) was used to determine the morphology and cross-sectional profile of the MNs. The material composition and crystallinity were characterized using EDX (AMETEK, Germany) and Raman spectroscopy (Renishaw InVia Raman microscope, UK). A Hysitron Triboindentor TI 950 (Bruker, US) was used to characterize the mechanical properties of the SMNs and CMNs. This measurement was performed using a Berkovich-shaped indentor. The bending and compression forces for the microneedle array were determined using an Instron microtester 5848 (Instron, US). Furthermore, biological insertion-based characterization for insertion of the needles in mouse skin was carried out on a customized platform. The protocol was approved with ethical clearance from the Committee for the Purpose of Control and Supervision of Experiments on Animals (CPCSEA), New Delhi, India. The aim of this step was to develop a cost-effective system of hollow CMNs to deliver drugs to the patient in a precise and painless manner. This process requires the combination of hollow MNs and flow channels that enable the drug from the reservoir to be delivered through the MNs. For this purpose, microfluidic conduits were etched in a silicon substrate using conventional potassium hydroxide (KOH) etching. Hollow MNs were fabricated after being properly aligned on these conduits. We also designed an in-house setup for microfluidic characterization. The CMN array on silicon was attached to a 5-ml syringe opening, and DI water was passed through the CMN array with varying pressure differences at the inlet. The test setup for flow rate characterization is shown in Fig. 6a, b. The pressure was monitored by an Abus PT series transducer.

## Conclusions

Carbon is an excellent choice of material for microneedle-based drug delivery applications. In this work, we present a fabrication approach for hollow CMNs, which are much stronger than their precursor counterpart, thus preventing any hazardous consequences arising from microneedle tip breakage. A laser source was used to pattern the MNs, and a pyrolysis process was applied for the conversion of SMNs to glassy CMNs. The entire conversion process was carried out at 900 °C in a N_2_ atmosphere.

We performed a parametric study to identify suitable microneedle dimensions for painless drug delivery. We selected 500 µm as the microneedle length and 100 µm as the outer diameter, while the inner diameter was varied from 40 µm to 90 µm in 10-µm steps. Structural shrinkage of the microneedle structure was observed and estimated using scanning electron microscopy. We observed that a hollow microneedle shape is retained in the structures. EDX results indicated that the carbon atomic percentage in the microneedle structure is ~ 94. However, the results also show the presence of oxygen and silicon due to the substrate (Si/SiO_2_) contribution. Raman spectroscopy results indicate that the microneedle structure is glassy in nature, which is biocompatible. The most important characterization performed in this work was the quantitative analyses of hardness and Young’s modulus using a nanoindentor. These analyses were carried out for both SMNs and CMNs, and the increases in hardness and Young’s modulus for the CMNs were estimated to be ~8- and 4.8-fold, respectively. A microneedle array was inserted into freshly excised mouse skin and withdrawn to examine the mechanical interaction of the MNs with skin. We found that the MNs penetrated the mouse skin surface and remained intact after retraction over multiple cycles.

Future work encompasses the development of a controlled drug delivery system including a micropump, a drug reservoir, and MNs, with the aim of developing a cost-effective system to deliver drugs to the patient in a precise and painless manner. We successfully fabricated microfluidic conduits in a silicon substrate and fabricated MNs aligned with the reservoir outlet.

## Electronic supplementary material

Supporting information
