# Supplementary material for: Glassy carbon microneedles—new transdermal drug delivery device derived from a scalable C-MEMS process
Source: Microsyst Nanoeng. 2018 Dec 17;4:38. doi: 10.1038/s41378-018-0039-9 (PMC6295442; doi:10.1038/s41378-018-0039-9)
Supplement: Supplementary file 1 — Supporting information [file 41378_2018_39_MOESM1_ESM.doc]

Glassy carbon microneedles – new transdermal drug delivery device derived from scalable C-MEMS process

Richa Mishra^a^, Bidhan Pramanick^b,@^, Tapas Kumar Maiti^c^, Tarun Kanti Bhattacharyya^d^

^a^Advanced Technology Development Center, IIT Kharagpur, West Bengal, India -721302; ^b^Department of Mechanical Engineering, IIT Kharagpur, West Bengal, India -721302; ^c^Biotechnology Department, IIT Kharagpur, West Bengal, India -721302; ^d^Department of Electronics and Electrical Communication Engineering, IIT Kharagpur, West Bengal, India -721302; @Current Address: School of Electrical Sciences, IIT Goa, Goa, India -403401

Correspondence: - Bidhan Pramanick (bpramanik@iitkgp.ac.in) or Tarun Kanti Bhattacharyya (tkb@ece.iitkgp.ernet.in)

**Table S1 Chemical composition determined by EDX for SU-8 and carbon microneedle**

| **Element** | **SU-8 microneedle**  **Atomic %** | **Carbon microneedle**  **Atomic %** |
| --- | --- | --- |
| Carbon | 76.35 | 94.46 |
| Nitrogen | 6.29 | - |
| Oxygen | 16.54 | 4.93 |
| Silicon | 0.82 | 0.61 |

**Table S2 Comparison of skin resistive forces and compressive and bending test results for SMN and CMN**

| **Type of MN** | **Type of Force** | **Resistive force offered by skin for chosen CMN (N)(Theoretical)** | **Magnitude of Force per CMN(N)** | **No. of times higher force offered by CMN** |
| --- | --- | --- | --- | --- |
| SMN (outer diameter 100μm, inner diameter 35 μm | Compression | 0.022 | 0.275 | 12.5 |
|  | Bending | 0.00022 | 0.012 | 54.54 |
| CMN (outer diameter 55μm, inner diameter 35 μm | Compression | 0.0044 | 1.84 | 418.18 |
|  | Bending | 0.000044 | 0.016 | 363 |

**Table S**3 Different geometries of fabricated microneedles

| **Sample Name** | **Outer Diameter SMN** | **Inner Diameter SMN** | **SU-8 MN wall thickness** |
| --- | --- | --- | --- |
| A | 100 | 40 | 30 |
| B | 100 | 50 | 25 |
| C | 100 | 60 | 20 |
| D | 100 | 70 | 15 |
| E | 100 | 80 | 10 |
| F | 100 | 90 | 5 |
